# Umbelliferone and scopoletin target tyrosine kinases on fibroblast-like synoviocytes to block NF-κB signaling to combat rheumatoid arthritis

**DOI:** 10.3389/fphar.2022.946210

**Published:** 2022-07-25

**Authors:** Qilei Chen, Wenmin Zhou, Yueming Huang, Yuanyang Tian, Sum Yi Wong, Wing Ki Lam, Ka Yee Ying, Jianye Zhang, Hubiao Chen

**Affiliations:** ^1^ School of Chinese Medicine, Hong Kong Baptist University, Hong Kong Special Administrative Region, Kowloon, Hong Kong SAR, China; ^2^ Guangzhou Municipal and Guangdong Provincial Key Laboratory of Molecular Target and Clinical Pharmacology, the NMPA and State Key Laboratory of Respiratory Disease, School of Pharmaceutical Sciences and the Fifth Affiliated Hospital, Guangzhou Medical University, Guangzhou, China

**Keywords:** rheumatoid arthritis, *Saussurea laniceps*, ultrafiltration, network pharmacology, ErbB network, drug discovery

## Abstract

Rheumatoid arthritis (RA) is a complex autoimmune condition primarily affecting synovial joints, which targeted synthetic drugs have damaging safety issues. *Saussurea laniceps*, a reputed anti-rheumatic medicinal herb, is an excellent place to start looking for natural products as safe, effective, targeted therapeutics for RA. Via biomimetic ultrafiltration, umbelliferone and scopoletin were screened as two anti-rheumatic candidates with the highest specific affinities towards the membrane proteomes of rheumatic fibroblast-like synoviocytes (FLS), the pivotal effector cells in RA. *In vitro* assays confirmed that the two compounds, to varying extents, inhibited RA-FLS proliferation, migration, invasion, and NF-κB signaling. Network pharmacology analysis and molecular docking analysis jointly revealed that umbelliferone and scopoletin act on multiple targets, mostly tyrosine kinases, in combating RA. Taken together, our present study identified umbelliferone and scopoletin as two major anti-rheumatic components from SL that may bind and inhibit tyrosine kinases and subsequently inactivate NF-κB in RA-FLSs. Our integrated drug discovery strategy could be valuable in finding other multi-target bioactive compounds from complex matrices for treating multifactorial diseases.

## Introduction

Rheumatoid arthritis (RA) is a chronic inflammatory joint disease that causes progressive articular destruction and deformity, and affects approximately 1% of the global population (Almutairi et al., 2021). Despite advances in RA management, the disease is still not well controlled in up to 30% of patients due to the individualized pathogenic network (McInnes and Schett, 2017). Current first-line RA medications include non-steroidal inflammatory drugs (NSAIDs) and disease-modifying anti-rheumatic drugs (DMARDs). These drugs can only relieve symptoms but fail to control disease progression (McInnes and Schett, 2011). Recently, highly targeted small-molecule agents, such as tyrosine kinase inhibitors (TKI), open a new avenue for RA therapy with improved clinical responses (Koenders and Van DenBerg, 2015). However, such synthetic molecules bring inevitable safety issues. For example, tofacitinib and baricitinib, two TKIs, have black box warnings from the US Food and Drug Administration (FDA) for severe infections and malignancies (Petronelli, 2021). The adverse events may not be correlated to specific protein inhibition but the synthetic structures (Schwartz et al., 2019). Therefore, novel scaffolds with safe, effective, targeted biomarker inhibition are desired for precision medicine in RA.

Natural products from herbal medicines have long been valuable sources for drug discovery due to their enormous scaffold diversity and structural complexity (Wang et al., 2021). The therapeutic rationale of herbal medicines is a paradigm of the emerging drug discovery concept of “polypharmacology”, *i.e.,* multiple components hitting multiple targets (Shen, 2015). Multi-targeted chemicals provide a better balance of efficacy and safety than single-targeted drugs in multifactorial diseases (Fang et al., 2019). “Snow lotus” has been reputed as an effective anti-rheumatic herbal medicine in Asia for centuries (Committee of Chinese Pharmacopoeia, 1995). We previously discovered that, among official “snow lotus” species (Chik et al., 2015; Fan et al., 2015; Chen et al., 2016), *Saussurea laniceps* Hand.-Mazz (SL) exerts the most outstanding chemical composition (Chen et al., 2017) and most potent anti-rheumatic efficacies (Yi et al., 2010, 2012), and SL significantly ameliorates RA symptoms by targeted inhibition of multiple therapeutic biomarkers while maintaining good safety profiles (Chen et al., 2021). Therefore, it is believed that SL is a promising source of safe, effective, targeted anti-rheumatic agents. However, therapeutic components in SL and corresponding action mechanisms have not been thoroughly investigated.

Isolation and activity screening of active compounds from herbal medicines, which are structurally diverse and sometimes in trace amounts, has long been challenging with existing conventional methods (Cannell, 1998). An advanced approach, bio-affinity ultrafiltration, based on receptor-ligand interaction, is a powerful tool for compound (ligand) fishing in complicated matrices due to its excellent high-throughput online screening ability, sensitivity, and selectivity (Chen et al., 2018). Generally, bio-affinity ultrafiltration uses one or a few recombinant cytosolic proteins as receptors (Wang et al., 2018). As one step further, a “biomimetic ultrafiltration” approach can be designed by employing natural disease-specific proteomes as receptors, which is more reasonable for screening active agents from complex matrices to treat multifactorial diseases, where most of the therapeutic targets remain highly underexplored (Fang et al., 2019).

Fibroblast-like synoviocytes (FLS) are the most common cell type at the pannus–cartilage junction and are critical effector cells in RA (Bustamante et al., 2017). A significant hallmark of FLS activation attributes to increased expression of surface proteins under inflammatory conditions (Wu Z et al., 2021). Therapies that target FLS, especially against their surface markers, are emerging as promising therapeutic tools for RA (Nygaard and Firestein, 2020). Therefore, proteins extracted from FLS of RA patients (HFLS-RA), including membrane and cytosolic proteomes, can be used as receptors in a tailored biomimetic ultrafiltration, to discover anti-rheumatic compounds from SL. The screened compounds are expected to hit multiple and even possibly unknown targets with minimized unspecific bindings. Such parallel study of compounds targeting natural proteomes from membrane and cytosol has not been reported.

In the present study, biomimetic ultrafiltration with FLS proteome fractions was conducted, followed by ultra-performance liquid chromatography coupled to a quadrupole/time-of-flight-mass spectrometer (UPLC-QTOF-MS) analysis, to fish out anti-rheumatic candidate compounds from SL. *In vitro* pharmacological performances of the screened compounds were verified, in terms of inhibiting RA-FLS proliferation, migration, invasion, and NF-κB activation. Protein targets and action pathways of the screened compounds were identified by network pharmacology analysis. Interaction modes between the compounds and key protein targets were investigated by molecular docking analysis. Our work provides valuable insights into the pharmacodynamic material basis of SL. It also serves as a case study to develop new drugs from natural resources for RA and other complex diseases.

## Materials and methods

### Chemicals and reagents

The standard compounds of umbelliferone, scopoletin, and celecoxib were purchased from Biomart Biotechnology Ltd. (Beijing, China). Acetonitrile (ACN) and methanol of chromatography grade were purchased from Lab-scan (Bangkok, Thailand). All aqueous solutions were prepared using ultra-pure water with a Milli-Q water purification system from Millipore (MA, United States). All chemicals not otherwise mentioned were purchased from Sigma-Aldrich (MO, United States) and were used without further purification.

### Plant material and extract preparation

SL was collected from Lhasa, Tibet, in 2008. The plant was authenticated by Prof. Hubiao Chen. Voucher specimens were deposited in the Hong Kong Baptist University. The aerial parts of the plant were powdered with a Fargo RT-04 grinder (Century Equipment Ltd., Kowloon, HK) and passed through a 20 mesh (0.9 mm) sieve. The dried and powdered sample (0.5 g) was reflux extracted with 25 ml water at 100°C for 1 h, twice. Total extracts were combined into a 100-ml volumetric flask and added up to the calibration mark with water. The extract was centrifuged at 14,000 *g* for 10 min before passed through a 0.2-µm syringe membrane (Alltech, IL, United States of America).

### Cell culture

Normal HFLS (HFLS-N) and HFLS-RA were purchased from Otwo Biotech Co., Ltd (Shenzhen, China) and Guandao Biological Engineering Co., Ltd (Shanghai, China), respectively. The cells were separately cultured in Dulbecco’s Modified Eagle’s medium (DMEM; Gibco, MD, United States) containing 10% (v/v) heat-inactivated fetal bovine serum (Gibco), 100 U/mL penicillin and 100 mg/ml streptomycin (Gibco). Cells were grown in a humidified atmosphere with 5% CO_2_ at 37°C.

### Biomimetic ultrafiltration coupled with UPLC-QTOF-MS analysis

HFLS-N and HFLS-RA cells were collected and washed three times with PBS (pH 7.4) with 1 mM phenylmethylsulfonyl fluoride (PMSF). The isolation of cell membrane and cytosol proteins was conducted using the Membrane and Cytosol Protein Extraction Kit (Beyotime, Shanghai, China) following the manufacturer’s instructions. Protein contents were determined by the Bradford method.

The screening procedure was conducted based on previous reports with modifications (Chen et al., 2018). Extracted proteins (2 mg/ml) and SL extract (5 mg/ml) were mixed in PBS buffer (pH 7.4) and rotated under 4°C for 30 min. To intercept the ligand–protein complexes, the mixtures were ultracentrifuged through filters (Molecular weight cut-off: 3 kDa; Amicon, Darmstadt, Germany) at 14,000 *g* under 4°C for 10 min, then washed with PBS four times. To release ligands, the obtained complexes were incubated with 500 μl MeOH for 10 min, then ultracentrifuged at room temperature for 15 min thrice. The combined filtrates were collected for chemical analysis. Heat-inactivated proteins were used as negative controls.

A UPLC-QTOF-MS system (Agilent Technologies, United States) was used for chemical analyses of SL extract and the ligand filtrates. The chromatographic and spectrometric parameters were set as previously published (Chen et al., 2017, 2021), with minor modifications regarding the elution gradient (solvent A: 0.1% formic acid in water; solvent B: 0.1% formic acid in ACN): 0–2 min with 2% B; 2–10 min with 2–10% B; 16–20 min with 18–25% B; 20–25 min with 25–55% B.

Affinity degree (AD) of a ligand towards an extracted proteome = (A_1_—A_2_)/A_0_ × 100%, where A_1_, A_2_, and A_0_ represented the peak areas of selected compounds obtained from the incubations of the SL extract with active, inactive, and without respective proteins, respectively.

RA- and membrane-specificity index = (AD_RM_/AD_NM_)/(AD_RC_/AD_NC_), where AD_RM_, AD_NM_, AD_RC_, and AD_NC_ represented the AD towards proteome from HFLS-RA membrane, HFLS-N membrane, HFLS-RA cytosol, and HFLS-N cytosol, respectively.

### MTT assay

Proliferation rate of HFLS-RA cells was monitored by an MTT assay as previously described (Chen et al., 2021). Briefly, cells (3  ×  10^4^/well) in 100 μl complete culture medium were seeded in 96-well microtiter plates for 24 h. They were then exposed to varying doses of celecoxib (positive control), umbelliferone, and scopoletin in serum-free medium. After incubation for 24 and 48 h, MTT (5 mg/ml, 10 μl) was added to each well, and the plates were incubated for 2 h. Formazan crystals were dissolved with 100 μl DMSO, and absorbance at 570 nm was measured by a Benchmark microplate reader (Bio-Rad, Hercules, CA, United States) with 630 nm as reference filter.

### Wound healing assay

Collective migration of HFLS-RA cells was assessed by a scratch wound healing assay as reported (Li et al., 2019). Cells (3  ×  10^5^/well) in 2 ml complete culture medium were seeded in 12-well microtiter plates for 24 h to reach 80% confluence. A linear wound was made in the cellular monolayer with a sterile 200 µl pipette tip. After removing the cell debris, the cells were incubated with 20 µM celecoxib, umbelliferone, and scopoletin in serum-free medium. Wound closures were observed and photographed at 0, 24, and 48 h under a light microscope (Leica DMI3000B, Wetzlar, Germany). The wound area was determined using ImageJ software.

### Transwell migration and invasion assay

24-well Transwell chambers (8 μm pore size; Corning, NY, United States) were used as published (Huang H et al., 2021). For single-cell migration assessment, HFLS-RA cells (4  ×  10^4^/well) in 200 μl serum-free medium were seeded in the upper chambers and treated with 20 µM celecoxib, umbelliferone, and scopoletin, respectively. 600 μl complete culture medium was added in the lower chambers. After incubation at 37°C for 24 h, non-migrating cells on the upper surface of the Transwell membrane were removed; cells migrated to the lower surface of the membrane were fixed with methanol, stained with 0.5% crystal violet, photographed under the microscope, and counted. The invasion assay was performed similarly, except that the membranes were pre-coated with Matrigel.

### Western blot analysis

HFLS-RA cells treated with 20 µM celecoxib, umbelliferone, and scopoletin, respectively, for 24 h, were collected and lysed using RIPA buffer (containing phosphatase inhibitor and PMSF) to obtain protein samples. An equal amount of protein was separated by 10% SDS-PAGE and transferred to PVDF membranes (Millipore). The membranes were blocked with 5% non-fat milk for 1 h at room temperature, and then incubated overnight at 4°C with the following primary antibodies: anti-NF-κB p65 (#8242; CST, MA, United States), phospho-NF-κB p65 (#3033; CST), IκBα (#4814; CST), phospho-IκBα (#2859; CST), and anti-GAPDH (#AF7021; Affinity, OH, United States). Then, the membranes were washed with 3 × TBST and incubated with corresponding HRP-conjugated secondary antibodies for 1 h at room temperature. The blots were developed using the enhanced chemiluminescence solution and imaged by ChemiDoc™ XRS + system.

### Network pharmacology analysis

To predict protein targets of umbelliferone and scopoletin, the two compounds were input in the form of Simplified Molecular Input Line System (SMILES) into SwissTargetPrediction database (http://www.swisstargetprediction.ch) and Search Tool for Interactions of Chemical (STITCH; http://stitch.embl.de); targets with literature support after checking at Uniprot (https://www.uniprot.org) were retained for subsequent analysis. RA-related genes were collected using GeneCards database (https://www.genecards.org) and Therapeutic Target Database (TTD, http://db.idrblab.net/ttd). Candidate targets related to both umbelliferone/scopoletin and RA were selected using a Venn diagram.

To conduct gene ontology (GO) and Kyoto Encyclopedia of Genes and Genomes (KEGG) pathway enrichment analysis, the selected targets were imported into the Database for Annotation, Visualization and Integrated Discovery (DAVID; https://david.ncifcrf.gov). The annotations with adjusted *p* < 0.05 were considered significantly enriched.

For each compound, compound–target–pathway (C-T-P) networks were constructed using Cytoscape (https://cytoscape.org) to examine the relationships between the compound, corresponding protein targets, and related pathways; protein–protein interaction (PPI) networks were generated using STRING (https://string-db.org) and visualized by Cytoscape to present the relationships between major protein targets of the compound.

### Molecular docking

Discovery Studio Biovia 2019 (Dassault Syst`emes, OH, United States), AutoDock Vina (The Scripps Research Institute, CA, United States), and PyMol (DeLano Scientific LLC, CA, United States) were employed for receptor/ligand structure modification, docking, 3D visualization, respectively. The crystal structures of the receptors were obtained from the Protein Data Bank (PDB; https://www.rcsb.org). All water molecules were removed, hydrogen polarities were assigned, and Gasteiger charges were computed. For each receptor, a grid was created to ensure the binding site covers the active pocket.

### Statistical analysis

Data are presented as the mean ± SD. Comparisons were performed using ANOVA tests unless specified. *p* < 0.05 was considered significant.

## Results

### Umbelliferone and scopoletin are ligands of RA-FLS membrane proteome

**FIGURE 1:**
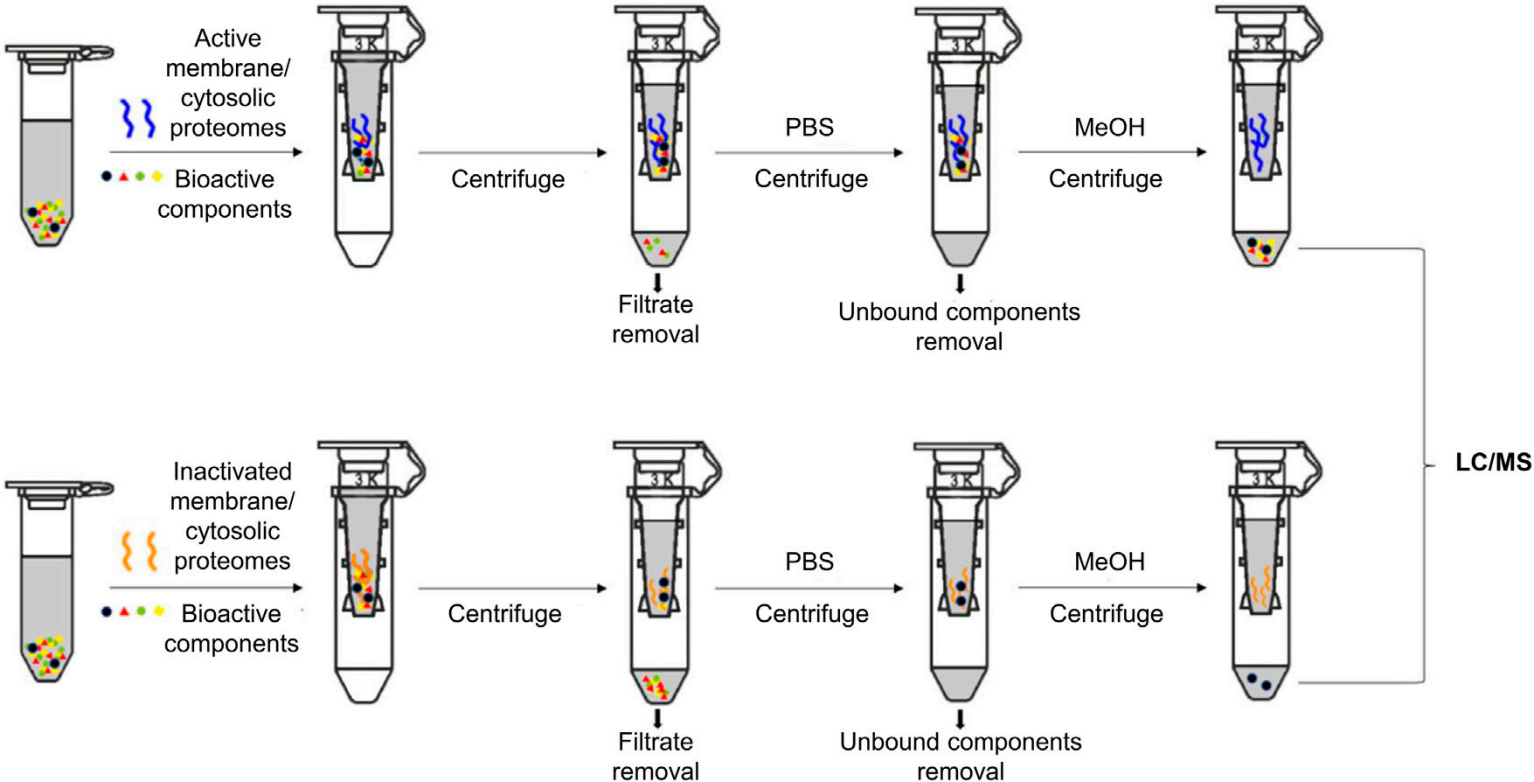
Schematic diagram of biomimetic ultrafiltration coupled with LC/MS.

Ligand fishing from the anti-rheumatic SL extract was based on biomimetic ultrafiltration coupled with UPLC-QTOF-MS analysis. Membrane and cytosolic protein fractions were employed as receptors in the ultrafiltration. To eliminate false-positive ligand fishing results, inactivated HFLS-RA-derived proteins were used as activity-negative control, and HFLS-N derived proteins as disease-negative control (Figure 1).

**FIGURE 2:**
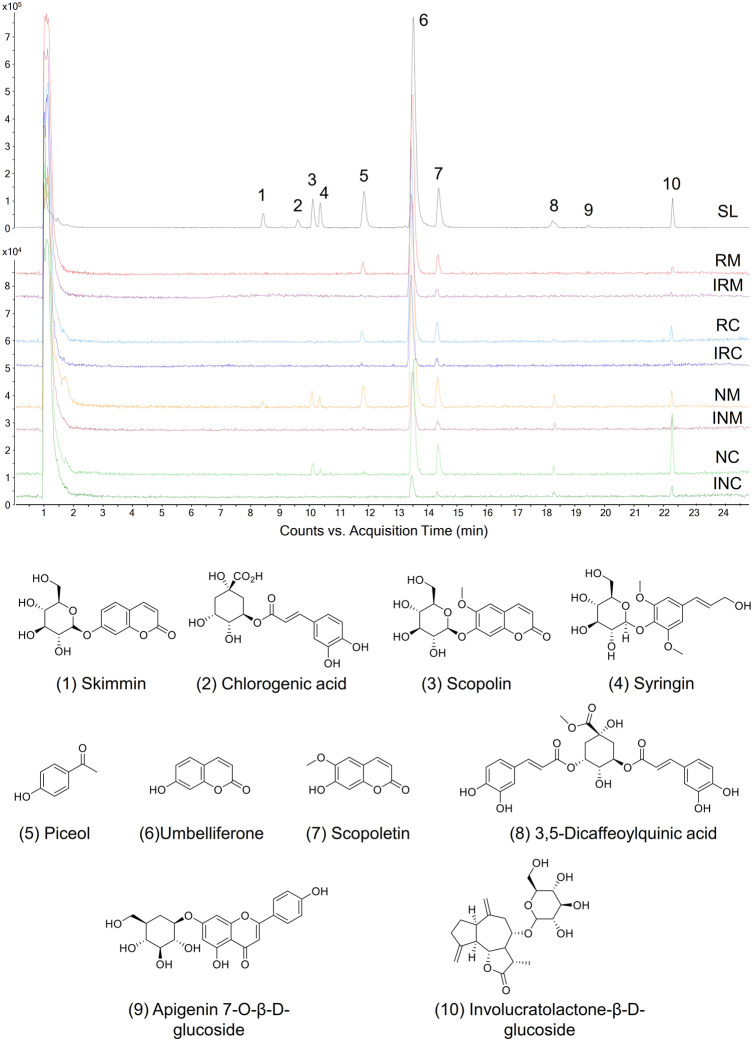
Proteome ligands separated from SL extract by biomimetic ultrafiltration coupled to UPLC-QTOF-MS. RM, RC: membrane and cytosolic proteomes of HFLS-RA, respectively. NM, NC: membrane and cytosolic proteomes of HFLS-N, respectively. IRM, IRC, INM, INC: inactivated corresponding proteomes, respectively.

Based on our previous chemical profiling of SL, ten major components were detected from the herbal extract (Figure 2) (Chen et al., 2017). Among the extract components, three compounds showed a AD towards rheumatic membrane (RM) proteins than that towards normal membrane (NM) proteins, namely, umbelliferone (peak 6; AD_RM_ = 25.82%; AD_NM_ = 10.27%), scopoletin (peak 7; AD_RM_ = 1.55%; AD_NM_ = 1.42%), and involucratolactone-β-D-glucoside (peak 10; AD_RM_ = 1.08%; AD_NM_ = 1.05%) (Table 1). Four compounds exerted higher AD towards rheumatic cytosol (RC) than normal cytosol (NC) fraction, namely, skimmin (peak 1; AD_RC_ = 3.40%; AD_NC_ = 1.88%), chlorogenic acid (peak 2; AD_RC_ = 0.10%; AD_NC_ = 0%), piceol (peak 5; AD_RC_ = 2.76%; AD_NC_ = 0.88%), and 3,5-dicaffeoylquinic acid (peak 8; AD_RC_ = 0.63%; AD_NC_ = 0%).

**TABLE 1:** UPLC-QTOF-MS data and proteome affinity degrees of SL extract components.

Peak	t_R_ (min)	Compound	Formula	Major fragment		AD_RM_ (%)^a^	AD_NM_ (%)	AD_RC_ (%)	AD_NC_ (%)	RA- and membrane-specific index
				*m*/*z*	Adduct ion					
1	8.43	Skimmin	C_15_H_16_O_8_	369.0893	[M + HCOO]^-^	0.31 ± 0.62	0.43 ± 0.58	3.40 ± 1.46	1.88 ± 0.13	0.40
2	9.59	Chlorogenic acid	C_16_H_18_O_9_	399.1004	[M-H]^-^	0.15 ± 0.01	0.26 ± 0.25	0.10 ± 0.13	0	/^b^
3	10.11	Scopolin	C_16_H_18_O_9_	399.0933	[M + HCOO]^-^	0.04 ± 0.06	0.51 ± 0.24	2.13 ± 1.33	2.78 ± 1.57	0.10
4	10.35	Syringin	C_17_H_24_O_9_	417.1402	[M + HCOO]^-^	0.34 ± 0.28	0.60 ± 0.32	1.99 ± 1.21	2.83 ± 0.21	0.81
5	11.84	Piceol	C_8_H_8_O_2_	135.0481	[M-H]^-^	1.03 ± 1.51	1.06 ± 0.93	2.76 ± 1.54	0.88 ± 0.13	0.31
6	13.56	Umbelliferone	C_9_H_6_O_3_	161.0244	[M-H]^-^	25.82 ± 12.03	10.27 ± 9.45	10.64 ± 6.17	18.09 ± 2.33	4.27
7	14.38	Scopoletin	C_10_H_8_O_4_	191.0372	[M-H]^-^	1.55 ± 1.99	1.42 ± 1.13	2.00 ± 2.08	6.54 ± 1.02	3.57
8	18.22	3,5-Dicaffeoylquinic acid	C_25_H_24_O_12_	515.1195	[M-H]^-^	0.38 ± 0.46	0.98 ± 0.60	0.63 ± 0.62	0	/^b^
9	18.73	Apigenin 7-O-β-D-glucoside	C_21_H_20_O_10_	577.1550	[M-H]^-^	1.60 ± 0.27	3.60 ± 2.43	8.03 ± 4.28	10.72 ± 2.77	0.59
10	22.31	Involucratolactone-β-D-glucoside	C_21_H_30_O_8_	455.2004	[M + HCOO]^-^	1.08 ± 1.10	1.05 ± 2.04	0	16.66 ± 2.12	/^b^

Data as mean ± SD (*n* = 3).

a AD_RM_, AD_NM_, AD_RC_, and AD_NC_, represented affinity degrees (AD) towards proteome from HFLS-RA, membrane; HFLS-N, membrane; HFLS-RA, cytosol, and HFLS-N, cytosol, respectively.

b Not applicable.

Cell surface proteins play crucial physiological roles *in vivo* and are currently the most successful class of drug targets for pharmaceuticals (Huang Y et al., 2021). In this regard, specific RA- and membrane-targeting compounds will be selected as high potential anti-rheumatic agents. Two coumarins from SL extract exhibited outstanding RA- and membrane-specificity index, *i.e.* umbelliferone (peak 6; index = 4.27) and scopoletin (peak 7; index = 3.57) (Table 1). Therefore, umbelliferone and scopoletin were screened from SL extract as key ligands of RA-FLS membrane proteome for subsequent analyses.

### Umbelliferone and scopoletin differentially inhibited RA-FLS activities

**FIGURE 3:**
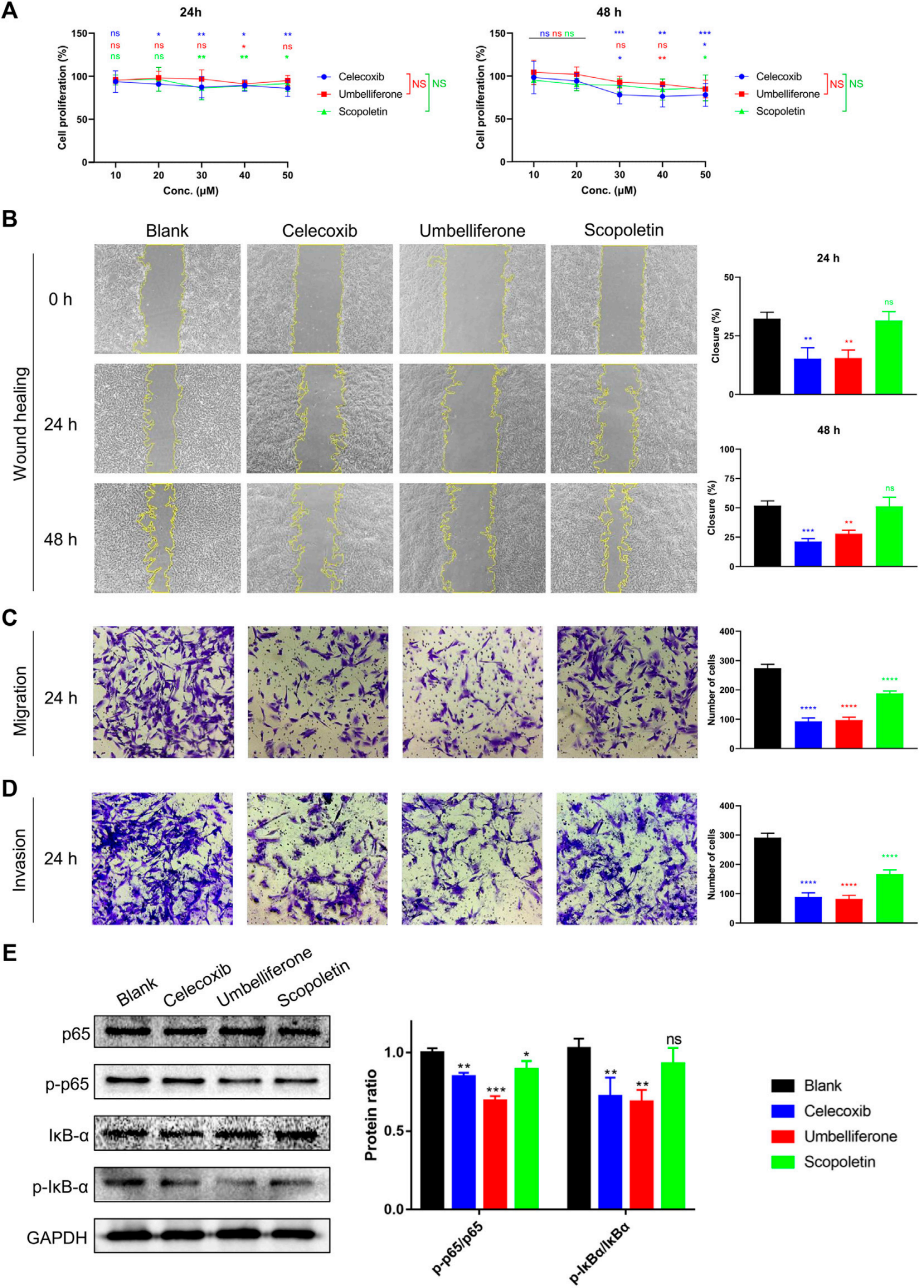
Umbelliferone and scopoletin differentially inhibited RA-FLS activities. **(A)** Proliferation, **(B)** wound healing, **(C)** Transwell migration, **(D)** Transwell invasion, and **(E)** western blot assays on HFLS-RA. Data are means ± SD of independent experiments performed in triplicate. ns, not significant at *p* > 0.05, *p < 0.05, ***p < 0.001, and ****p < 0.0001 vs blank control group; ns, not significant vs celecoxib group.

Inhibitory effects of the two compounds on RA-FLS activities were assessed regarding proliferation, migration, and invasion. An MTT study showed that umbelliferone and scopoletin shared similar inhibitory profiles on HFLS-RA proliferation with celecoxib, a first-line RA drug yet associated with increased cardiovascular risks (Figure 3A) (Fidahic et al., 2017). Specifically, scopoletin significantly inhibited HFLS-RA proliferation at 30 μM and onwards. In our previous research, umbelliferone and scopoletin were proved with evidently lower cardiomyocyte toxicity and less cardiac remodeling in rats than celecoxib did (Chen et al., 2021). Our previous and current findings collectively indicate that the two screened natural compounds are safer for the cardiovascular system yet match competitiveness in inhibiting RA-FLS proliferation compared to celecoxib.

For inhibition of HFLS-RA migration, umbelliferone showed similarly potent efficacies with celecoxib and was more effective than scopoletin. At 20 μM, where the dosage was proved with no significant impact on cell proliferation, umbelliferone and celecoxib significantly reduced both single-cell and collective migration of HFLS-RA cells with 24 and 48 h incubation (*p* < 0.0026) (Figures 3B,C). Scopoletin exhibited significant reduction of single-cell migration (*p* < 0.0001) but only slight inhibition of collective migration (*p* ≤ 0.8919) (Figures 3B,C). Since cell-extracellular matrix (ECM) adhesion and cell-cell adhesion are central mechanisms in the process of collective migration (Pijuan et al., 2019), our results indicated that umbelliferone is more potent in inhibiting such adhesions than scopoletin.

The compounds’ inhibitory effects on HFLS-RA invasion were also evaluated (Figure 3D). Umbelliferone exerted significant (71.82% inhibition; *p* < 0.0001) and even more evident reduction of invasive cells than celecoxib (69.42% inhibition; *p* < 0.0001). Scopoletin also reduced the number of invasive cells, yet the inhibitory rate was approximately half that of umbelliferone. The inhibitory pattern of invasion being similar to that of collective migration can be explained that the two processes share similar cell behaviors: they both involve cells with mesenchymal characteristics to degrade the ECM for directed cell movements (Friedl and Gilmour, 2009; Yang et al., 2019).

The transcription factor NF-κB is a pivotal mediator of inflammatory responses. NF-κB activation is associated with RA-FLS hyperproliferation, migration, and invasiveness, leading to hyperplasia in the rheumatic synovium (Nejatbakhsh Samimi et al., 2020). We hereby investigated whether the tested compounds inhibit the NF-κB signaling pathway. The western blot results indicated that the compounds decreased the phosphorylation of NF-κB p65 and IκB-α, paralleling their inhibitory levels (Figure 3E). Such suppressed NF-κB canonical signaling can decrease the expression of inflammatory cytokines, adhesion molecules, and other promoters in the inflammatory cascade (Liu et al., 2017). The conclusion of the two compounds suppressing NF-κB signaling can be supported by other studies showing that umbelliferone (Ouyang et al., 2019; Wu G et al., 2021) and scopoletin (Li et al., 2009; Chen et al., 2021) can effectively suppress phosphorylation of specific NF-κB subunits (e.g. p65 and IĸBα) and expression of downstream genes (e.g. IL-1β, TNF-α, MMP-3, MMP-9, COX-2, Bcl-2) in RA synovial tissues and FLSs. Since both umbeliferone and scopoletin had high binding affinities towards RA-FLS membrane proteins (Table 1) and inhibited RA-FLS activities (Figure 3), it is believed that NF-κB is a primary node of the two compounds in treating RA.

### Umbelliferone and scopoletin target key pathways and proteins against RA

**FIGURE 4:**
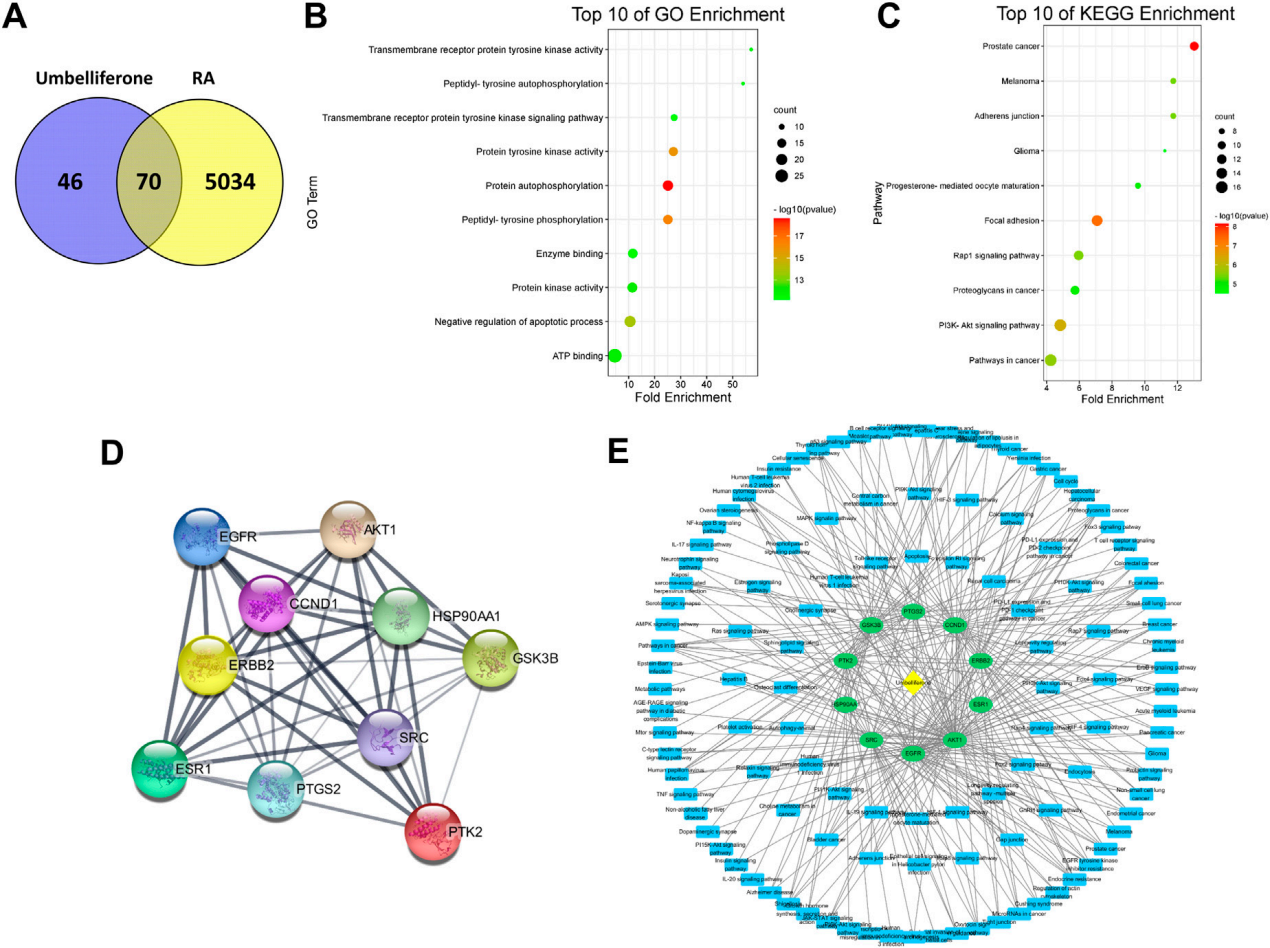
Network pharmacology analysis of umbelliferone treating RA. **(A)** Venn diagram of umbelliferone and RA-related targets. **(B)** The top ten GO enrichment. **(C)** The top ten KEGG enrichment. **(D)** PPI network showing top ten protein targets. **(E)** C-T-P network based on top ten protein targets.

**FIGURE 5:**
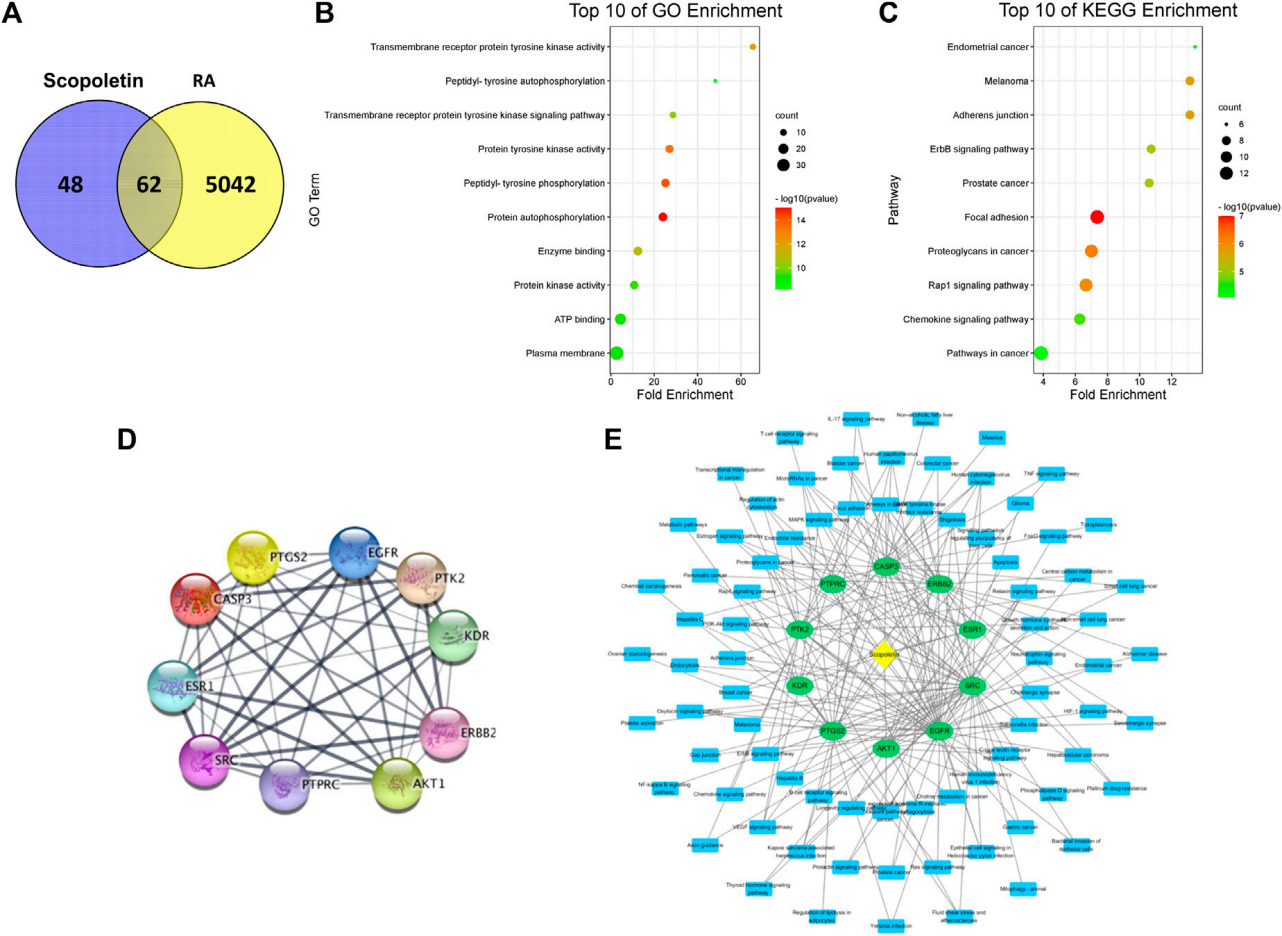
Network pharmacology analysis of scopoletin treating RA. **(A)** Venn diagram of scopoletin and RA-related targets. **(B)** The top ten GO enrichment analysis. **(C)** The top ten KEGG enrichment analysis. **(D)** PPI network showing top ten protein targets. **(E)** C-T-P network based on top ten protein targets.

A network pharmacology analysis was conducted to identify direct biological targets of umbelliferone and scopoletin in treating RA. From various databases, 5104 RA-related proteins, 116 umbelliferone protein targets, and 110 scopoletin protein targets were collected with literature support. It is worth noticing that over half of the protein targets of the two screened compounds are linked with RA (Figure 4A and Figure 5A), *i.e.,* 70 (60.34%) proteins and 62 (56.36%) proteins for umbelliferone and scopoletin, respectively, corresponding to their abovementioned promising anti-rheumatic efficacy. The overlapping proteins were recognized as candidate anti-rheumatic targets of the two respective compounds (Supplementary Tables S1, S2).

C-T-P networks were constructed for the two compounds. The C-T-P network of umbelliferone consisted of 70 targets and 117 pathways, and that of scopoletin included 62 targets and 109 pathways (Supplementary Figures S1, S2). On such basis, Gene Ontology (GO) and KEGG pathway enrichment analysis were employed to characterize the functional annotations of the proteins (Supplementary Figures S3, S4). Interestingly, umbelliferone and scopoletin shared highly similar GO and KEGG profiles. According to the GO enrichment results, the two compounds are both heavily involved with transmembrane receptor tyrosine kinases (RTKs), especially with their activities (GO: 0004714, 0004173, 0004672), phosphorylation (GO: 0038083, 0046777, 0018108), and signaling pathways (GO: 0007169) (Figure 4B and Figure 5B). As for the KEGG results, adherens junction (KEGG: hsa04520), focal adhesion (KEGG: hsa04510), and Rap1 signaling pathway (KEGG: hsa04015) are among the ten most significant pathways for both compounds (Figure 4C and Figure 5C). Apart from the shared KEGG enrichments, umbelliferone is particularly enriched in phosphatidylinositol 3-kinase (PI3K)-protein kinase B (Akt) signaling pathway (KEGG: hsa04151), while scopoletin particularly in signaling pathways of ErbB (ErbB; KEGG: hsa04012) and chemokines (KEGG: hsa04062). Therefore, it is predicted that both umbelliferone and scopoletin can act on TKs to regulate biological processes, including cell adhesion, cell-cell junction formation, and cell polarization, which are all critical during RA progression (Weyand and Goronzy, 2021).

PPI networks for umbelliferone and scopoletin were also analyzed (Supplementary Figures S5, S6). There were seven common proteins among the top ten targets of the two compounds, namely, epidermal growth factor receptor (EGFR/ErbB1), proto-oncogene tyrosine-protein kinase Src (Src), Akt serine/threonine kinase 1 (Akt1), receptor tyrosine-protein kinase ErbB-2 (ErbB2/HER2), protein tyrosine kinase 2 (PTK2/FAK), prostaglandin-endoperoxide synthase 2 (PTGS2/COX-2), and estrogen receptor 1 (ESR1/ERα) (Figure 4D and Figure 5D). Most of the listed proteins can be located on cell membrane; many of them can be categorized as RTKs (e.g., EGFR and ErbB2), non-receptor TKs (e.g. Src and FAK), or proteins closely crosstalking with TKs (e.g. Akt1 and ERα). Simplified C-T-P networks were constructed based on umbelliferone and scopoletin’s top ten protein targets, respectively (Figure 4E and Figure 5E). Specifically, the present network pharmacology analysis results correspond with our previous study in which the two compounds exerted significant and selective COX-2 inhibition in rheumatic rat synovium tissues (Chen et al., 2021).

**FIGURE 6:**
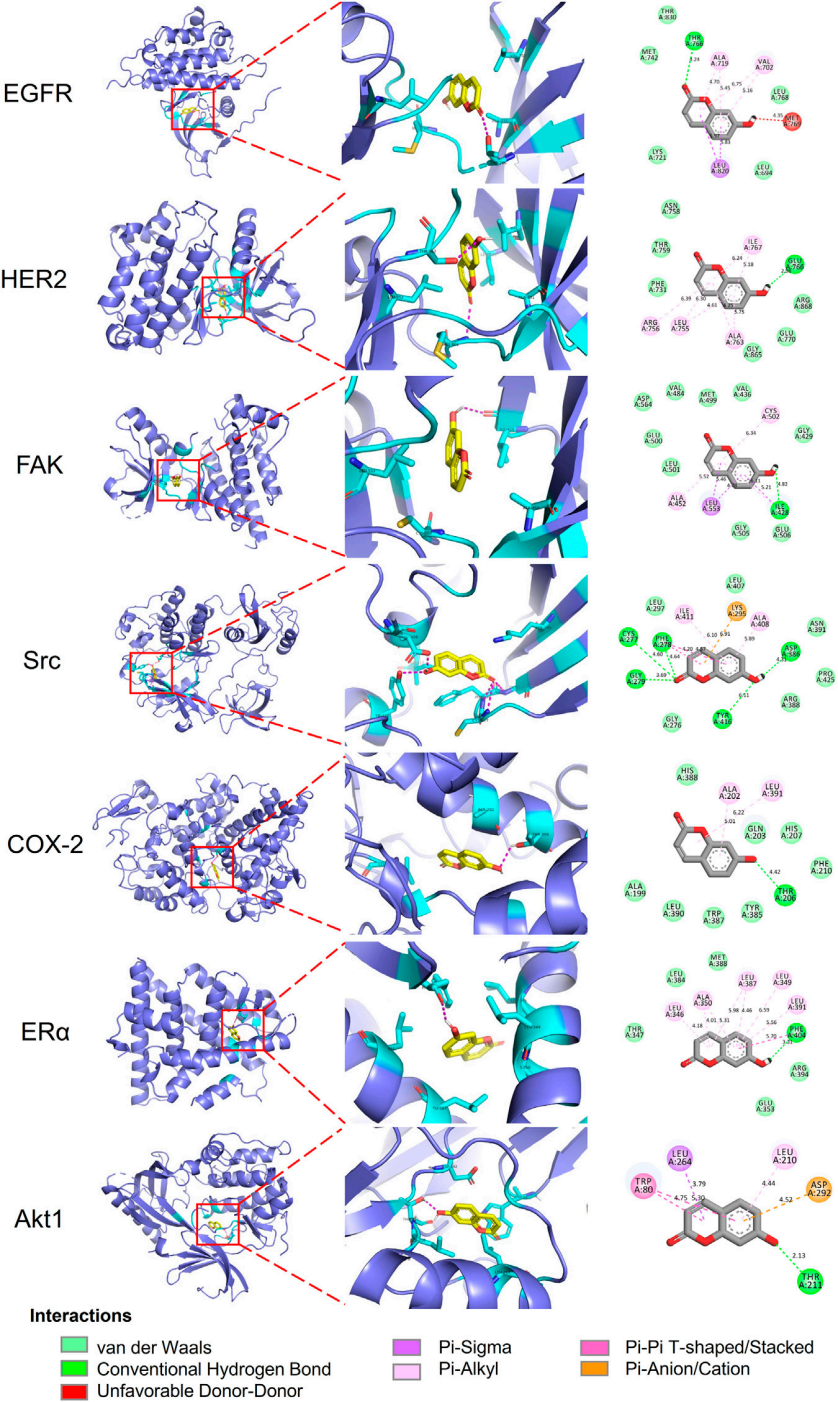
Docking patterns of umbelliferone with selected top target proteins. 3D residues in cyan indicate representative ligand–receptor interactions. Numbers indicate bond distance (Å).

**FIGURE 7:**
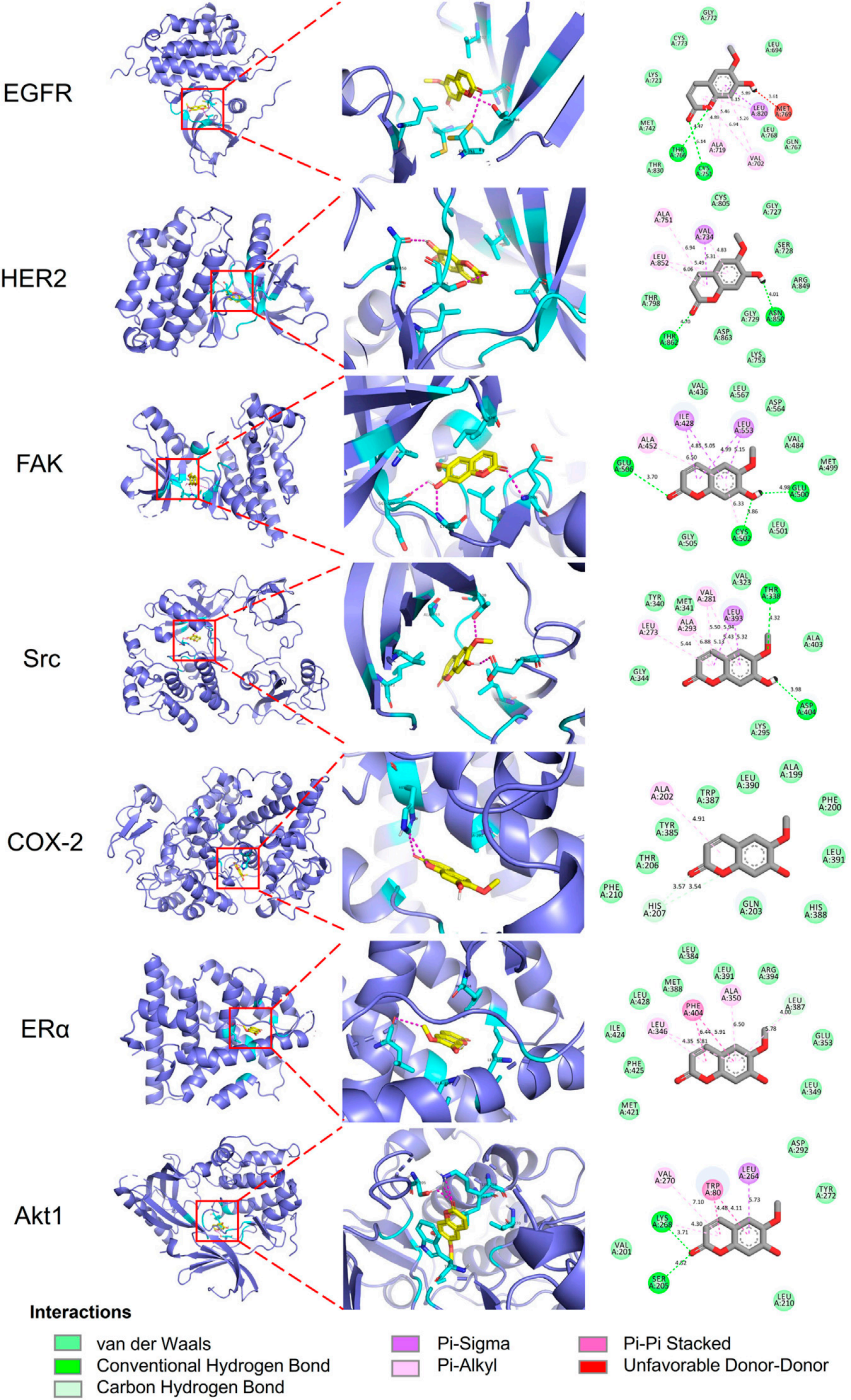
Docking patterns of scopoletin with selected top target proteins. 3D residues in cyan indicate representative ligand–enzyme interactions. Numbers indicate bond distance (Å).

Molecular docking analyses of umbelliferone and scopoletin against the abovementioned seven common top protein targets were performed to investigate the ligand-receptor interactions (Figures 6, 7). The docking studies were all validated by redocking the co-crystalized ligand for each protein, where the root mean square deviation (RMSD) value below 2Å was considered good solutions (Supplementary Figure S7) (Ramírez and Caballero, 2018). Generally, the analyzed proteins all showed satisfactory affinities towards the two compounds (Supplementary Table S3). Among the analyzed TKs, HER2 is the protein exhibiting the lowest binding energies towards umbelliferone (-6.9 kcal/mol) and scopoletin (-7.2 kcal/mol), respectively. All interacting residues of HER2 with both compounds lie in the active pocket. The interactions are mainly via conventional H-bonds (umbelliferone: Met_801_, Thr_862_; scopoletin: Asn_520_, Thr_862_), π-alkyl bonds (umbelliferone: Leu_726_, Val_734_, Ala_751_, Leu_852_; scopoletin: same except without Leu_726_), and π-σ bonds (umbelliferone: Val_734_, Leu_852_; scopoletin: Val_734_). Collectively, the molecular docking results indicate favorable interactions and binding mechanisms between the two compounds and their top target proteins.

## Discussion

### Umbelliferone and scopoletin act on FLSs by targeting TKs and blocking NF-κB signaling

**FIGURE 8:**
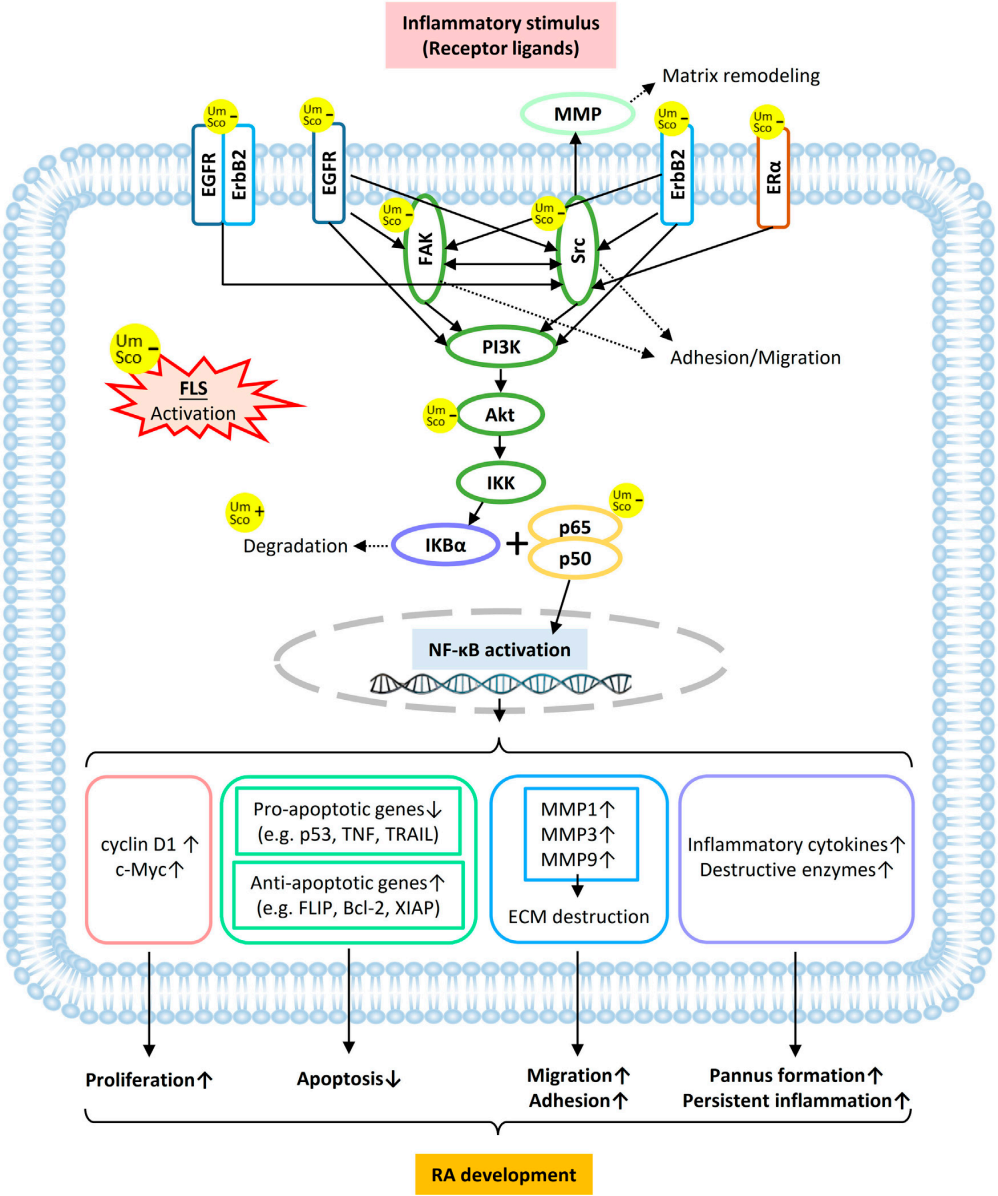
Schematic overview of umbelliferone and scopoletin targeting membrane and cytosolic proteins in RA-FLS in attenuating RA development *via* NF-κB blockade.

According to our present study, in treating RA, umbelliferone and scopoletin 1) exert high binding affinities towards membrane proteins of RA-FLSs, 2) directly target TKs (mostly membrane-bound) and proteins with close interaction with TKs, 3) inhibit NF-κB signaling in RA-FLSs, and 4) attenuate RA-FLS activities (Figures 2–5). A schematic overview of the signaling network regarding the two compounds inhibiting FLS activation is drawn, featuring main protein targets shared by the two compounds, such as EGFR, ErbB2, Src, FAK, ERα, and Akt (Figure 8). In the ErbB signaling network, ErbB family members, including EGFR, ErbB2, and their heterodimers, signal through Src and FAK to activate a myriad of downstream signaling pathways (Yarden and Pines, 2012). Src-induced tyrosine phosphorylation of FAK is a central mediator of focal adhesion turnover and cell migration (Wu et al., 2015). ErbB RTKs, Src, and FAK activate the PI3K/Akt cascade to affect diverse cellular functions, including chemokine signaling and endocrine resistance. Akt also regulates NF-κB signaling to induce target gene expression (Liu et al., 2020). In the estrogen signaling network, stimulated ERα not only forms a complex with Src and PI3K, leading to Akt activation, but also activates Src, which in turn enhances matrix metalloproteinase (MMP) expression to facilitate ECM destruction (Pan et al., 2020). In summary, umbelliferone and scopoletin inhibit NF-κB activation in RA FLSs mainly via the ErbB/PI3K/Akt signaling axis.

NF-κB activation in RA-FLSs can lead to a series of cancerous and inflammatory features of the synoviocytes (Figure 8). The activation of NF-κB induces the expression of cyclin D1 and c-Myc, which are cell growth promoters, subsequently boosting cell proliferation. Various anti-apoptotic signals are delivered, including inhibition of pro-apoptotic genes (e.g., p53 activity loss due to p65 Ser536 phosphorylation) and increased expression of anti-apoptotic genes (e.g. FLIP in the Fas/FasL pathway). Since gene promoters of most MMPs have canonical sites for NF-κB, activated RA-FLSs secret elevated levels of MMPs, resulting in increased invasiveness and cartilage erosion. IκB kinase (IKK) can be stimulated by inflammatory cytokines (e.g. IL-2 and TNF-α) and further mediate the inflammatory signaling cascade (Ghosh and Karin, 2002; Liu et al., 2017). In addition, FLS-induced RA pathogenesis switches on inflammatory responses from various immune cells in the RA synovium, such as dendritic cells, lymphocytes, and monocytes, which further maintain FLS activation and perpetuate the disease progression (Nejatbakhsh Samimi et al., 2020). Therefore, it is believed that the anti-rheumatic effects of umbelliferone and scopoletin can be at least partly attributed to the blockage of NF-κB signaling via targeting TKs on activated FLSs.

### Umbelliferone and scopoletin targeting RA-FLS membrane raises hope to combat RA

Membrane proteins perform a myriad of biological functions, including cell communication, substance transportation, and catalytic reactions. Such proteins on the cell surface account for over 60% of the targets of all FDA-approved small-molecule drugs (Huang Y et al., 2021). As FLS are the critical effector cells in RA, FLS membrane proteins, such as transmembrane TKs, play pivotal roles in RA progression through orchestrating cellular signaling among different cell types (Kovács et al., 2014). However, despite the significance, drug discovery against membrane proteins is notoriously challenging, mainly because 1) membrane proteins are difficult to be isolated with maintained structure and functions, and 2) this category of proteins is still largely under-investigated. Our present study demonstrates an integrated drug discovery strategy, using SL herb as an example, featuring biomimetic ultrafiltration to screen compounds targeting RA-specific membrane proteins. As a result, our strategy enables the discovery of safe, effective compounds hitting intact, multiple membrane targets with minimized unspecific bindings to combat RA. Therapeutic agents discovered by this strategy are promising advantages compared with current first-line drugs for RA, e.g. NSAIDs and DMARDs, that lack clinical responses because 1) selectively inhibiting multiple and possibly novel targets, e.g., TKs, helps to control RA as a heterogeneous disease, 2) minimizing unspecific bindings can prevent adverse events of some current synthetic targeted drugs (Koenders and Van DenBerg, 2015).

There have been reports on anti-rheumatic performances of umbelliferone and scopoletin. On a rat model with Freund’s complete adjuvant (FCA)-induced arthritis, umbelliferone was found to reduce pro-inflammatory cytokines, such as TNF-α and IL-1β (Ouyang et al., 2019; Wu G et al., 2021), and osteoclastogenesis biomarkers, such as MMP-3 and MMP-9 (Wu G et al., 2021); according to the authors, such reduced gene expressions were due to suppressed NF-κB signaling upon umbelliferone administration. On the same rat model, scopoletin significantly alleviated clinical symptoms, immune responses, and joint pathological conditions (Chen et al., 2021); scopoletin was also proved to induce RA FLS apoptosis by inhibiting NF-κB activation (Li et al., 2009). Despite such findings, neither umbelliferone nor scopoletin has reached routine clinical therapy for RA. One possible reason might be a lack of confirmation on their direct target proteins and subsequent pivotal signaling axis. Our current study fills this gap by elucidating that membrane proteins on FLSs, especially TKs, are among the direct targets of umbelliferone and scopoletin; the two compounds may function via the RTK/PI3K/Akt axis to reach inhibition of NF-κB and downstream cascades.

### Drug discovery for natural resource preservation

Drug discovery and development from herbal medicines that are derived from rare herbal species can significantly preserve natural resources. There is little cultivation of SL; almost all SL material in commerce has been collected from the wild. In the Himalayan region, due to the reputed anti-rheumatic potency of SL, the population vitality and survival of the species are threatened by heavy and illegal harvesting of the plant (Chen et al., 2016). Since umbelliferone and scopoletin are the two most abundant and therapeutically potent components in SL, precise elucidation of action mechanisms of the two compounds enables the development of synthetic analog drugs, which can, in turn, effectively save the endangered situation of SL.

## Conclusion

Taken together, our present study demonstrates three main steps in elucidating therapeutic components and corresponding action mechanisms in the anti-rheumatic herb SL. Firstly, umbelliferone and scopoletin were screened from SL as two compounds with the highest specific binding affinities towards RA-FLS membrane proteins. Secondly, reduced activities of RA-FLSs and decreased NF-κB activation were confirmed under the administration of either umbelliferone or scopoletin. Thirdly, the two compounds are identified with crucial target proteins (including TKs) and pathways (including RTK/PI3K/Akt/NF-κB) against RA. Therefore, we can conclude that umbelliferone and scopoletin, as major active ingredients from SL, can target tyrosine kinases on FLSs to block NF-κB signaling in attenuating progression of RA. Our study not only contributes to elucidating the multi-component and multi-target anti-rheumatic mechanisms of the endangered species of SL but also helps develop safe, effective anti-rheumatic drugs based on chemical scaffolds of umbelliferone and scopoletin.

## Data Availability

The original contributions presented in the study are included in the article/Supplementary Materials, further inquiries can be directed to the corresponding authors.
