# Supplementary material for: Umbelliferone and scopoletin target tyrosine kinases on fibroblast-like synoviocytes to block NF-κB signaling to combat rheumatoid arthritis
Source: Front Pharmacol. 2022 Jul 25;13:946210. doi: 10.3389/fphar.2022.946210 (PMC9358226; doi:10.3389/fphar.2022.946210)
Supplement: Supplementary file 1 [file DataSheet1.docx]

Supplementary Material

# Supplementary Figures

**Supplementary Figure 1.** C-T-P network of umbelliferone treating RA.

**Supplementary Figure 2.** C-T-P network of scopoletin treating RA.

**Supplementary Figure 3.** GO enrichment analysis of umbelliferone treating RA. BP: biological process; CC: cellular component; MF: molecular function.

**Supplementary Figure 4.** GO enrichment analysis of scopoletin treating RA. BP: biological process; CC: cellular component; MF: molecular function.

**Supplementary Figure 5.** PPI network of umbelliferone treating RA, showing 70 targets.

**Supplementary Figure 6.** PPI network of scopoletin treating RA, showing 62 targets.

**Supplementary Figure 7.** Superimposition of the poses of the redocked ligands (yellow) and the co-crystalized ligand (cyan) within selected target proteins.

# Supplementary Tables

**Supplementary Table 1.** Candidate anti-rheumatic targets of umbelliferone.

| Gene official symbol | |  |  |
| --- | --- | --- | --- |
| AKT1 | HSP90AA1 | SRC | EGFR |
| ESR1 | ERBB2 | CCND1 | PTGS2 |
| GSK3B | PTK2 | FYN | SNCA |
| KDR | LYN | PARP1 | CDK4 |
| CCNA2 | IGF1R | HSPA1A | NFKB1 |
| MET | HDAC6 | PDGFRB | FGR |
| ESR2 | CDK2 | ACHE | HDAC2 |
| COMT | CDK5 | NQO1 | PTK2B |
| GSR | BRAF | AKR1B1 | CNR1 |
| CXCR1 | INSR | MPO | PLK1 |
| CYP1A2 | MAOA | FLT4 | TYR |
| RPS6KA3 | TEK | XDH | MIF |
| MB | CA1 | CSNK2A1 | CA2 |
| PTPN22 | CA3 | IDO1 | CYP2D6 |
| ALDH2 | EPHB4 | MAP3K8 | ERN1 |
| KCNMA1 | TNNT2 | KCNA3 | SRD5A1 |
| QDPR | ALPL | PLAA | METAP2 |
| CA4 | RNASEH1 |  |  |

**Supplementary Table 2.** Candidate anti-rheumatic targets of scopoletin.

| Gene official symbol | |  |  |
| --- | --- | --- | --- |
| EGFR | GSK3B | BRAF | SRC |
| IGF1R | CASP3 | PTK2 | MET |
| ERBB2 | INSR | PTGS2 | PDGFRB |
| AKT1 | UGT1A8 | UGT1A6 | UGT1A1 |
| PIK3CG | LYN | KDR | MAOA |
| CYP1A2 | FLT4 | ESR1 | CSNK2A1 |
| TEK | ESR2 | ALOX5 | XDH |
| TERT | MITF | F2 | CXCR1 |
| COMT | CA2 | AOC3 | PTPRC |
| PTPN1 | PLK1 | MAP3K8 | HMGCR |
| CA4 | CA1 | ALPL | WEE1 |
| PARP1 | GRK6 | CA3 | SRD5A1 |
| PNP | KCNMA1 | GSR | FGR |
| EPHB4 | PLAA | MB | KCNA3 |
| HSPA1A | CHRNA7 | ALPG | AKR1B1 |
| ADRA2C | ACHE |  |  |

**Supplementary Table 3.** Calculated binding energies of umbelliferone and scopoletin within active sites of selected target proteins.

| Target protein | PDB code | Bing energy (kcal/mol) | |
| --- | --- | --- | --- |
|  |  | Umbelliferone | Scopoletin |
| EGFR | 1M17 | -6.3 | -6.4 |
| HER2 | 3PP0 | -6.9 | -7.2 |
| FAK | 3BZ3 | -6.5 | -6.7 |
| Src | 2SRC | -6.8 | -6.7 |
| COX-2 | 1CX2 | -7.9 | -7.6 |
| ERα | 1R5K | -6.8 | -6.4 |
| Akt1 | 3O96 | -7.0 | -7.3 |
